# Editorial Comment: Prediction Model for Neurogenic Bladder Recovery One Year After Traumatic Spinal Cord Injury

**DOI:** 10.1590/S1677-5538.IBJU.2023.06.02

**Published:** 2024-02-07

**Authors:** Andrés G. Alfieri, Marcio A. Averbeck

**Affiliations:** 1 Hospital Italiano de Buenos Aires Buenos Aires Argentina Hospital Italiano de Buenos Aires, Buenos Aires, Argentina; 2 Hospital Moinhos de Vento Unidade de Videourodinâmica Porto Alegre RS Brasil Chefe de Neuro-Urologia, Unidade de Videourodinâmica, Hospital Moinhos de Vento. Porto Alegre, RS, Brasil

Sally El Sammak 1, Giorgos D Michalopoulos 1, Namrata Arya 2, Archis R Bhandarkar 3, F M Moinuddin 1, Ryan Jarrah 1, et al.

**World Neurosurg. 2023;S1878-8750(23)01166-X.**

**DOI: 10.1016/j.wneu.2023.08.054 | ACCESS: 37611802**

## COMMENT

El Sammak S. et al. have aimed to build a prediction model for neurogenic lower urinary tract dysfunction (NLUTD) recovery 1 year after traumatic spinal cord injury (SCI). A multivariable odds logistic regression model was applied to the National Spinal Cord Injury Model Systems (NSCIMS) database, which prospectively collects demographic, functional, medical, and other outcomes of people with SCI. From 2000 to 2016, 2515 patients with NLUTD after acute SCI were identified, of whom 417 patients (16.6%) recovered bladder function within 1 year. Patients aged 30-44 made up the majority of the population (26.5%). Most patients (79.8%) were male, and the three most frequent traumatic etiologies were violence (11.7%), falls (28.4%), and car accidents (43.3%). Sacral sensation, American Spinal Injury Association (ASIA) impairment score, bowel function at baseline, voluntary sphincter contraction, anal sensation, S1 motor scores, and the number of days in the rehabilitation facility were identified as predictors of bladder recovery, with a discriminative capacity of 90.5%.

For the purpose of maximizing patient care, managing patient expectations, and promoting individualized rehabilitative efforts, it is essential to understand the trajectory of bladder recovery in SCI patients. This prediction model may ultimately help us counsel our patients through the first year of rehabilitation and provide good arguments in favor of assisted bladder emptying options. Although this prediction model provides relevant information, one should emphasize the role of invasive urodynamics to assess the risk of upper urinary tract deterioration among patients with traumatic SCI, particularly for those with suprasacral lesions. According to the European Association of Urology (EAU) guidelines on Neuro-Urology, urodynamic investigation should be performed as a mandatory baseline diagnostic intervention and at regular intervals in high-risk patients (1).
